# A Review on Remediation Technology and the Remediation Evaluation of Heavy Metal-Contaminated Soils

**DOI:** 10.3390/toxics12120897

**Published:** 2024-12-10

**Authors:** Lei Xu, Feifei Zhao, Xiangyu Xing, Jianbiao Peng, Jiaming Wang, Mingfei Ji, B. Larry Li

**Affiliations:** 1 Henan Province Engineering Research Center of Environmental Laser Remote Sensing Technology and Application, Nanyang Normal University, Nanyang 473001, China; terry__xp@163.com; 2 Collaborative Innovation Center of Water Security for Water Source Region of Mid-Line of South-to-North Diversion Project of Henan Province, Nanyang Normal University, Nanyang 473001, China; 3 Non-Major Foreign Language Teaching Department, Nanyang Normal University, Nanyang 473001, China; xxyxxy880902@163.com; 4 College of Water Resources and Modern Agriculture, Nanyang Normal University, Nanyang 473001, China; pjb126com@163.com (J.P.); wangjmecology@163.com (J.W.); jimfdy@gmail.com (M.J.); 5 Ecological Complexity and Modeling Laboratory, Department of Botany and Plant Sciences, University of California–Riverside, Riverside, CA 92521, USA; bai-lian.li@ucr.edu; 6 International Joint Laboratory of Watershed Ecological Security and Collaborative Innovation Center of Water Security for Water Source Region of Middle, Nanyang Normal University, Nanyang 473001, China

**Keywords:** soil, heavy metal contamination, remediation technology, remediation standard, remediation evaluation

## Abstract

With the rapid development of industry and agriculture, soil contamination has become a significant environmental issue, and the heavy metal contamination of soils is an important part of it. The main methods for the remediation of heavy metal-contaminated soils include physical methods, chemical methods, biological methods, and combined remediation methods have been proposed as research deepens. However, the standards and evaluation methods for the remediation of heavy metal-contaminated soils are still not well-established. This article discusses the sources and contamination status of heavy metals in soils, the advantages and disadvantages of remediation technology for heavy metal-contaminated soils, remediation standards, and post-remediation evaluation methods. It also proposes scientific issues to be addressed in future research and provides an outlook on future development, hoping to assist in subsequent remediation studies of heavy metal-contaminated soils.

## 1. Introduction

As one of the natural and essential resources for human survival, the soil bears the burden of providing food and other renewable resources for humanity. However, with the development of industries, agricultural production, and the advancement of modern technology, heavy metal contamination, especially soil heavy metal contamination, has become one of the major issues impairing global environmental quality as well as human survival and development [1]. The characteristics of soil contamination, such as its concealment, long-term nature, and irreversibility, coupled with the difficulty in degrading heavy metal elements by soil microorganisms, mean that, once the soil is contaminated by heavy metals, restoring its original ecological functions will consume a significant amount of manpower and resources [2,3]. Therefore, the remediation and improvement of heavy metal-contaminated soils have become an important research area in soil science, ecology, and environmental science. In recent years, the remediation of heavy metal-polluted soils has become a hot topic of research [4,5]. However, the establishment of standards and evaluation for the remediation of heavy metal-contaminated soils are influenced by a variety of factors, such as the background values of the soil, the technological methods used in the remediation process, and ecological toxicological assessments of the contamination. This creates an awkward situation of how to clearly define the extent of soil remediation that can be considered as clean and the level at which it will not pose a threat to subsequent reuse. Therefore, conducting this work scientifically and orderly can further promote the steady development of soil remediation work.

This study focuses on analyzing the sources and hazards of heavy metals in soils, the remediation methods for heavy metal-contaminated soils, the standards for the remediation of heavy metal-contaminated soils, and the evaluation after remediation. Through this research, feasible plans for the future remediation of heavy metal-contaminated soils will be provided, and a methodological basis for establishing a systematic study of contaminated soil remediation benchmarks will be offered, promoting the establishment of an ecological security system.

## 2. Sources and Hazards of Soil Heavy Metals

The sources of heavy metals in soils are extensive, and in addition to being related to natural factors such as the parent material, soil formation processes, and volcanic activities, human activities are the most important sources of soil heavy metal pollution [6,7]. Irrigation with wastewater is a feasible way to solve the water needs of crops in arid areas, but improper irrigation measures and the introduction of a large amount of wastewater exceeding heavy metal standards have caused serious pollution to agricultural soils [8,9]. Mining and smelting industrial processes generate a large amount of heavy metal elements, and some enterprises directly discharge untreated waste into the environment, causing nearby soils to be enriched with heavy metals [10,11]. Erin LeGalley used isotope tracing to study street sediments in Ohio and found that the sediments contained a large amount of heavy metals such as Cu, Cr, Co, and Pb, which mainly come from the yellow lines on the roads and automobile exhaust [12]. The issue of soil heavy metal contamination caused by the long-term application of organic and chemical fertilizers is also increasingly receiving attention. Studies have found that organic fertilizers and phosphate fertilizers contain higher levels of heavy metals, and the long-term application of phosphate fertilizers is a major factor affecting the accumulation of heavy metals in soils [13,14,15]. Recent studies have increasingly found that atmospheric deposition is becoming a significant source of soil heavy metal contamination [16,17]. This is due to the fact that the heavy metals emitted into the atmosphere from activities such as fossil fuel combustion, vehicle exhaust, and non-ferrous mining and smelting can impact ecosystems and human health, both directly and indirectly, through multi-pathway transportation [18]. The typical sources of heavy metals in soils are listed in Table 1.

Soil heavy metal contamination can have a severe impact on ecosystem stability and human health. Firstly, soil heavy metal contamination can hinder the growth and development of crops, thereby reducing crop yield and quality [19]. For example, under the stress of high concentrations of Pb and Cd, wheat height decreases and secondary roots are reduced, ultimately leading to a decrease in wheat yield [20,21]. At the same time, Hg, Cd, and Pb are the most serious heavy metal elements exceeding standards in vegetables in suburban areas of our country [22,23]. Moreover, the accumulation of heavy metals in soil may also lead to changes in soil properties, thereby changing the supply of nutrient elements and fertility characteristics. For example, when there is an excessive accumulation of heavy metals in soil, the adsorption of soil phosphorus, the mineralization of organic nitrogen, and the change in the potassium form all hinder the retention and supply of N, P, and K in the soil, thereby affecting crop yield and quality [24,25,26]. Heavy metals exceeding a certain concentration have a significant impact on the activity and quantity of soil microorganisms [27]. Long-term positioning experiments have shown that, when the soil is contaminated by Zn, Cd, Cu, Ni, Pb, and Cr, the nitrogen-fixing activity of cyanobacteria can be reduced by 50%, and their numbers are also significantly reduced [28]. Studies have found that heavy metal stress affects soil enzyme activity [29], and soil urease activity decreases with the increase in mercury pollution concentration [30]. The difference in soil urease activity among different pollution levels is highly significant. When the amount of mercury added to the soil reaches 12mg/kg, the soil urease activity is only 34% compared to the control, indicating that soil urease is very sensitive to mercury pollution [31]. In addition, crops in heavy metal-contaminated soils will absorb and utilize heavy metals and partially enrich them in edible parts, entering the human body through the digestive tract via the food chain, causing a series of diseases such as cancer and hypertension [32]. The typical effects of heavy metals on ecosystems and human health are listed in Table 1.

**Table 1:** The sources and hazards of typical heavy metals in soils.

Types	Source	Major Existence Forms ^a^	Environmental Toxicity
Hg	Irrigation (60%), atmospheric deposition (35%), livestock and fertilizer (5%) [33]	Residual Hg (12.3%), organic-bound Hg (86.2%), oxidized Hg (1.5%) [34]	Affects seed germination and plant morphogenesis; causes sensory abnormalities, ataxia, delayed intellectual development, and language and hearing impairments in humans [35]
Cd	Irrigation (67%), atmospheric deposition (32%), livestock and fertilizer (1%) [33]	Exchangeable Cd (39.2%), carbonate-bound Cd (4.32%), Fe–Mn oxide-bound Cd (21.9%), organic matter-bound Cd (7.58%), residual Cd (27.0%) [36]	Disrupts the protein structure of soil microorganisms, causing them to die due to protein denaturation, resulting in soil compaction [37]; excessive intake of copper may cause acute and chronic poisoning in the human body [38]
Pb	Irrigation (13%), atmospheric deposition (84%), livestock and fertilizer (1%) [33]	Exchangeable Pb (33.4%), carbonate-bound Pb (13.2%), Fe–Mn oxide-bound Pb (13.8%), organic matter-bound Pb (9.55%), residual Pb (30.0%) [39]	Affects the physiological systems of the human body, including the urinary system, reproductive system, gastrointestinal system, endocrine system, and cardiovascular system [40]
As	Irrigation (71%), atmospheric deposition (20%), livestock and fertilizer (9%) [33]	Water-soluble As (5.25%), surface-adsorbed As (14.4%), iron–aluminum oxide-bound As (15.2%), acid-extractable As (4.95%), residual As (60.2%) [41]	Disrupts the structure of soil microbial communities [37]; causes acute or chronic poisoning, and can also induce skin cancer, lung cancer, and bladder cancer, etc. [42]
Zn	Irrigation (26%), atmospheric deposition (72%), livestock and fertilizer (2%) [33]	Exchangeable Zn (4.11%), carbonate-bound Zn (1.15%), Fe–Mn oxide-bound Zn (3.06%), organic matter-bound Zn (1.06%), residual Zn (90.6%) [43]	Excessive zinc can lead to disorders in the oxidative metabolism of myocardial cells, simple osteoporosis, and atrophy of the brain tissue [44]
Cu	Irrigation (62%), atmospheric deposition (35%), livestock and fertilizer (3%) [33]	Exchangeable Cd (20.7%), carbonate-bound Cd (10.6%), Fe–Mn oxide-bound Cd (14.6%), organic matter-bound Cd (18.8%), residual Cd (30.2%) [36]	It inhibits chlorophyll synthesis [45]; reduces the number of bacteria, fungi, and actinomycetes in the soil [46]; and decreases the activity of urease and nitrate reductase [47]
Cr	Irrigation (22%), atmospheric deposition (72%), livestock and fertilizer (6%) [33]	Exchangeable Cr (3.07%), carbonate-bound Cr (7.43%), Fe–Mn oxide-bound Cr (21.6%), organic matter-bound Cr (11.6%), residual Cr (56.3%) [36]	It affects the activity of soil urease and catalase; it induce respiratory diseases such as bronchial asthma; long-term contact with the skin can cause erythema, eczema, and other skin diseases; and it is highly likely to cause cancer [48]

^a^ There are various methods to evaluate the forms of metal elements present in soil. The method used in this study was the one commonly employed by many researchers. Additionally, the speciation distribution of heavy metal elements varies significantly across different soils. The data provided in this study are for reference only.

## 3. Remediation Technologies for Heavy Metal-Contaminated Soil

Based on current research, the remediation methods for heavy metal-contaminated soils mainly include physical remediation, chemical remediation, biological remediation, and combined remediation. Single physical and chemical remediation methods are costly and may cause secondary pollution, making them difficult to implement in large-scale soil remediation projects. As an emerging remediation technology, phytoremediation has the advantages of low cost, good effect, minimal environmental disturbance, and no destruction of landscape ecology, and has attracted much attention and application [49,50]. Combined remediation technology involves the combined application of two or three of physical, chemical, and biological remediation methods to achieve soil remediation and ecological restoration, and has a broad prospect in future application and research [51,52]. The commonly used heavy metal soil remediation technologies and the advantages and disadvantages of these technologies are listed in Table 2.

### 3.1. Physical Remediation

The physical remediation of heavy metal-contaminated soils refers to the use of single physical methods for soil improvement, aiming to reduce and stabilize heavy metals in the soil. Common methods include soil replacement, washing, solidification and landfilling, thermal treatment, and electrokinetic remediation. 

#### 3.1.1. Tilling and Soil Replacement

Tilling involves mechanically turning over the soil deeply to disperse and transfer heavy metals concentrated in the surface layer to deeper levels, thereby diluting them [67]. Soil replacement involves adding a large amount of clean soil to the contaminated soil and mixing it with the original soil to reduce pollutant concentrations below critical hazard levels [68]. Tilling and soil replacement are effective for treating lightly contaminated soils and can achieve the established remediation goals in the short term [53]. However, they require a significant amount of labor and resources, with high investment costs, and may reduce soil fertility and primary productivity, making them unsuitable for large-scale promotion [69].

#### 3.1.2. Thermal Treatment

High-temperature thermal treatment technology refers to the method of volatilizing some volatile heavy metals such as Hg and As from the soil by heating. This method can remove 99% of PAHs and volatile pollutants from the soil [70], and also has a certain fixation effect on some non-volatile heavy metals and radioactive elements, reducing their environmental risks [71,72]. However, high-temperature pyrolysis can destroy soil organic matter and structural water, and it requires a large amount of energy consumption, leading to limited practical application [73].

#### 3.1.3. Solidification

Solidification refers to the addition of stabilizing agents to heavy metal-contaminated soils to change its physical and chemical properties, reducing the mobility and bioavailability of heavy metals through adsorption and co-precipitation [74]. Stabilizing agents include single agents such as lime, cement, and fly ash, as well as composite stabilizing agents, which are mixed in various proportions to improve and enhance engineering technical performance. Solidified soils not only reduce the mobility and bioavailability of heavy metals but can also be used in building materials and roadbed construction, killing two birds with one stone [75]. However, solidification technology has inherent shortcomings, such as soil destruction, significant disturbance to the ecosystem, and the need for a large amount of stabilizing agents, leading to high costs. Therefore, it is only suitable for small-area pollution control, and further research is needed for large-area application.

### 3.2. Chemical Remediation

Chemical remediation is an in situ remediation technology that involves adding amendments or inhibitors to the soil to cause a series of chemical reactions in heavy metals, reducing their solubility, mobility, and bioavailability in the soil, thereby achieving the purpose of governance and remediation [76,77]. Currently, the application of amendments to the soil is more common, and the ideal amendment should have the characteristics of being pollution-free, low-cost, effective, and durable. Commonly used amendments such as lime, limestone powder, apatite, hydroxyapatite, and calcium–magnesium phosphate fertilizer have certain remediation effects on the soil. Surface-active agents and heavy metal chelating agents can also be added to achieve improvements.

Lime and carbonate minerals are among the most commonly used heavy metal immobilization materials. Studies have shown that adding 0.2% lime to the soil can reduce the available forms of Cu and Cd by 97% and 86%, respectively [78]. The main alkaline materials for remediating heavy metals include calcium carbonate (CaCO_3_) and dolomite [CaMg (CO_3_)_2_]. After adding lime or calcium magnesium phosphate to contaminated soils, the exchangeable form of Cd can be reduced significantly [79]. Phosphorus-containing materials are one of the most effective heavy metal immobilization agents, and the main phosphorus-containing immobilization materials used in soil heavy metal immobilization include apatite, calcium phosphate, superphosphate, and phosphorus-containing sludge [80,81,82]. Silicon-containing materials have certain remediation effects on heavy metals such as aluminum, iron, zinc, cadmium, and manganese [83]. Commonly used silicon-containing materials include silicate fertilizers, calcium silicate, silicon-containing sludge, fly ash, and silicate clay minerals [84]. Biochar is a carbon-rich material that has been utilized to remediate heavy metal-contaminated farmland. It has been increasingly used in the remediation of heavy metal-contaminated soils due to its multiple benefits, including reducing soil heavy metal contamination, improving soil fertility, and promoting soil carbon sequestration [85,86]. Its application in soil remediation has shown promising results in laboratory and field trials, and it is considered a cost-effective and environmentally friendly soil amendment. Meanwhile, utilizing the antagonistic effects of heavy metals in the soil environment can reduce their toxicity. For example, increasing the supply level of Ca can reduce the plant absorption of heavy metals, such as Cd, Ni, Pb, Zn, and Cr, or alleviate the toxicity of heavy metals [87]. Therefore, adding calcium-containing substances to the soil can also reduce the bioavailability of some heavy metals.

Chemical remediation is simple and easy to perform, and industrial by-products can be used as amendments during the remediation process, making the cost relatively low and suitable for large-area contaminated soil remediation [88]. However, single chemical remediation cannot completely remove heavy metals from the soil, and they may be released back into the soil when environmental conditions change [89]. It may also have certain impacts on soil microorganisms, posing potential safety hazards. Therefore, when applying chemical remediation, materials with stable effects and minimal impact on the soil ecosystem should be considered as amendments.

### 3.3. Bioremediation

Bioremediation is divided into broad and narrow senses. The bioremediation we usually refer to is the broad sense of bioremediation, which means using organisms as the main technology for the remediation of heavy metal contamination. Through their metabolic activities, it reduces the content of heavy metals in the soil or reduces their toxicity by changing their form, thus achieving the purpose of remediation. Common bioremediation technologies include phytoremediation, fauna remediation, and microbioremediation.

#### 3.3.1. Phytoremediation Technology

Phytoremediation technology refers to the use of plants and their rhizosphere microorganisms to extract, absorb, volatilize, transform, degrade, and fix pollutants in the soil, thereby removing pollutants from the soil [90]. The mechanisms of phytoremediation include phytoextraction, rhizodegradation, phytodegradation, phytostabilization, and phytovolatilization [91]. Among them, phytoextraction utilizes the absorption of heavy metals by certain hyperaccumulator plants, and by properly handling the harvested aboveground parts, it removes heavy metals from the soil, achieving the goal of reducing pollution and eliminating toxicity [92]. This technology is widely used in current research on the phytoremediation of heavy metal-contaminated soils.

As a technology for the remediation of heavy metal contamination, phytoremediation depends on several aspects: the degree of soil pollution, the effectiveness of rhizosphere microorganisms, and the ability of applied plants to intercept, absorb, concentrate, and degrade heavy metals [93]. Therefore, choosing the right plants is crucial in pollution remediation. Existing studies have shown that plants of the genus Alyssum have the ability to hyperaccumulate Ni [94]; plants of the genus Thlaspi have the ability to hyperaccumulate Cd [95]; Indian mustard (Brassica juncea) and corn show a hyperaccumulation of Pb under EDTA enhancement, with the Pb content in the aboveground parts of Indian mustard exceeding 15,000 mg/kg [96]; the highest concentration of As in the fronds of Pteris vittata L of the Pteridaceae family can reach 22,630 mg/kg, and the bioconcentration factor (BF) exceeds 10 [97]; and Sedum plumbizincicola is a hyperaccumulator of Cd and Zn [98]. Utilizing phytoremediation for heavy metal contamination also increases soil organic matter content and soil fertility, and the increase in surface vegetation cover is beneficial to the improvement in the ecological environment, and the cost is low [99,100]. Therefore, how to use biotechnology to cultivate new hyperaccumulator plants has become a hot topic in phytoremediation research.

#### 3.3.2. Soil Fauna Remediation

Narrowly defined, soil fauna refers to animals that spend their entire life history in the soil, while broadly defined, soil fauna are animals that come into contact with the soil surface or live in the soil during a certain period (or a certain season) of their life history. The soil fauna remediation technology we usually refer to is the use of broad-sense soil animals. It refers to the use of soil animals and their intestinal microorganisms to decompose, digest, and concentrate pollutants in polluted soil under artificially controlled or natural conditions, thereby reducing or eliminating pollutants, which is a kind of bioremediation technology [101]. The mechanisms of fauna remediation are roughly as follows: soil animals absorb heavy metals and form metallothioneins [102]; peptides produced through metabolism reduce the activity of heavy metals through chelation; under heavy metal stress, a variety of heavy metal transport protein genes are produced to enhance resistance [103]. Due to the limitations of fauna remediation, there is less research on it, and the current focus is on using earthworms, rodents, and other large soil animals for remediation. Its application in actual remediation projects requires further research.

#### 3.3.3. Microbial Remediation

Microbial remediation refers to the use of naturally occurring or specially cultured microorganisms to degrade organic pollutants in the soil under controllable environmental conditions, or to change the form of toxic elements through biosorption and bio-oxidation/reduction reactions, thereby reducing their toxicity and ecological risk in the environment [104,105]. The remediation mechanisms of different microorganisms vary greatly. Bacterial microorganisms mainly change the form of heavy metals, while fungi reduce the concentration of free heavy metal ions through chelation with metallothioneins in their bodies, thereby reducing their toxicity [106,107]. Microbial remediation technology is flexible and can be carried out in situ, ex situ, or a combination of in situ/ex situ remediation. Moreover, microbial remediation has the characteristics of low cost and high efficiency, and has become a hot topic in heavy metal remediation research in recent years [108]. However, there are many problems in the application of microbial remediation, such as the fact that the added microorganisms may not adapt to the environment or may not be able to survive due to competition with other microorganisms in the environment, and are greatly affected by environmental conditions [109]. Therefore, cultivating microorganisms with strong adaptability and broad-spectrum characteristics through modern biotechnology and applying them to pollution control will become a trend in environmental remediation research.

### 3.4. Combined Remediation Technology

Due to the increasing complexity of soil pollution and the significant differences in pollution levels, soil types, regional conditions, and reuse requirements in different areas, a single remediation method can no longer achieve the desired remediation effects. How to develop compound remediation technologies according to local conditions has become a direction in the research on the remediation of heavy metal-contaminated soils [110]. The commonly used types of combined remediation technologies are plant–microorganism-combined remediation, animal–plant-combined remediation, and physical–chemical–biological-combined remediation [111,112,113]. Plant–microorganism-combined remediation, as an intensified plant remediation technology, has gradually become a hot topic in domestic and international research [114]. According to the different forms of plant–microorganism-combined remediation of heavy metal-contaminated soils, this combined remediation technology can be divided into two forms: plant-combined remediation with specific strains and plant-combined remediation with mycorrhizae [115]. Like other remediation methods, plant–microorganism combined remediation technology is also affected by many factors during application, such as the degree of soil heavy metal pollution, the availability of heavy metals, the characteristics of plants themselves, and changes in the rhizosphere environment (pH, redox potential, rhizosphere secretions, rhizosphere microorganisms, and rhizosphere minerals) [116,117,118,119,120].

## 4. Remediation Standards and Evaluation of Heavy Metal-Contaminated Soil

The establishment of remediation and evaluation standards for heavy metal-contaminated soils, from the perspective of ecological toxicology or human health protection, has become a key component in the remediation of heavy metal pollution in some developed countries. Most developed countries regulate the environmental management of contaminated sites through legislation, forming a legal system and management system, and have developed a relatively complete system of remediation technologies through a large number of research and practices in remediation [121,122]. However, due to various factors, there is still relatively little research in this area in China to date. Establishing regional- or even national-level evaluation standards through scientific research and demonstration will have significant practical implications for China’s agricultural product safety, ecosystem health, and sustainable development of land resources, and should be of high concerned by government departments and environmental workers.

### 4.1. Remediation Standards for Heavy Metal-Contaminated Soil

In the process of formulating remediation standards for heavy metal-contaminated soils, it is necessary to fully consider factors such as the level of economic development, social factors, clean technology factors, regulatory factors, and political factors [123]. Therefore, the remediation standards vary greatly among countries, and even different provinces or states within the same country have significantly different soil standards. Table 3 lists the remediation standards for heavy metal contaminated soils in different countries and regions. For example, New Jersey’s “Soil Remediation Standards” in the United States classify land resource remediation standards into three categories: residential land, non-residential land, and remediation standards for potential impacts on groundwater. Different land types have corresponding pollutant types, health risk standards, and remediation standards [124]. Denmark’s soil standards are quite unique; different from general soil standards, they are not based on a comprehensive basis such as groundwater and atmospheric standards for soil standard setting, but are based on the differences in soil regions, according to soil quality benchmarks, pollution area pollutant reduction standards, pollution area groundwater quality standards, and pollution area air quality standards for the establishment of soil remediation standards [125]. The Netherlands established a brand-new set of soil environmental standards based on human health and ecological risk research after discontinuing its original soil and groundwater standards in 1994. The new standards can effectively indicate the pollution of soil and groundwater, aiming to build the country’s ultimate soil quality goals [126]. China’s soil environmental quality standards (GB15618—2018) classify soil environmental quality into three categories based on the application function and protection objectives of the soil, which to some extent provide a basis for soil pollution remediation work in China [127]. However, due to the large number of soil types in China, the significant differences in properties, varying degrees of pollution in different regions, and factors such as composite pollution, it cannot fully solve the problems in the actual work. More importantly, the standards do not focus on people and do not propose standards related to places where people live, such as residential areas, commercial areas, and industrial areas, which are closely related to human health and sustainable development; so, they still need to be further improved.

The establishment of remediation standards will be affected by various factors, such as soil background values, technical methods used in remediation, the instrumental detectable level of pollutants, the adjustability of standards, and ecological toxicological evaluation [131,132]. Therefore, in the process of formulating standards, the existing classification methods should not be limited. The ultimate utilization objectives of soil remediation should be fully considered, integrating the issues of reuse after soil remediation and the risks to human health [133]. It is essential to formulate soil remediation standards that are suitable for different regions, types, and levels of pollution, tailored to local conditions [134].

### 4.2. Remediation Evaluation of Heavy Metal-Contaminated Soil

In the remediation work of heavy metal-contaminated soils, observing the soil after remediation and evaluating it through scientific methods can clarify whether the remediated soil meets the standards, whether the threat of pollution to human health and the ecosystem has been eliminated, and whether it can be reused. This is the final and even the most critical step. There are many evaluation methods for heavy metal contaminated soil remediation, and Table 4 lists the commonly used evaluation criteria.

#### 4.2.1. Post-Remediation Monitoring of Contaminated Soil

The post-remediation monitoring of contaminated soils is to observe the damage to different biological components in the soil ecosystem after remediation, and to qualitatively or quantitatively evaluate the risk of the remediated soils to the ecosystem and human health [140]. According to the difference in monitoring sites, it can be divided into laboratory monitoring and in situ monitoring. According to the restoration target function recovery indicators, it can be divided into biochemical toxicological monitoring and ecological indicator monitoring. According to the difference in monitoring time, it can be divided into short-term monitoring and long-term monitoring [141,142,143]. Laboratory monitoring is to collect soil samples from the remediation site and conduct ecological toxicological monitoring indoors. Laboratory monitoring has certain limitations and is therefore affected in its application, while in situ monitoring is to evaluate the remediation effect through the ecological toxicological diagnosis of the remediation site, which can more objectively and comprehensively evaluate the remediation effect [144].

The primary goal of remediating contaminated soil is to restore its function as a habitat (plants and soil animals). Ecological indicator monitoring is based on this goal, introducing some sensitive organisms (plants, soil animals, and microorganisms) into the remediated soil, and judging the effect of soil remediation by monitoring their physiological and ecological changes [145]. There are several methods: plant symptom and growth method; sensitive animal indicator method; biomarker method; broad bean root tip micronucleus test method; and soil enzymology indicator method [146,147,148,149,150]. Ecological indicator monitoring can intuitively identify the ecological safety of soil pollutants, closely linking the cleanliness of the soil and the effect of remediation, and has certain practicality in the evaluation of contaminated soil remediation. Biochemical toxicological monitoring is based on the potential hazards of residual pollutants in the soil to the physiological and biochemical aspects of the environment and evaluates the size of the risk through molecular- or cellular-level measurements, thereby evaluating the remediation effect [137]. This method has the advantages of high measurement sensitivity and short measurement cycle.

#### 4.2.2. Ecological Risk Assessment of Reused Remediated Soil

Risk assessment is a process that predicts the possibility of pollutants in the environment having adverse ecological effects on the entire ecosystem or parts of it [151]. Soil ecological risk assessment focuses on the potential impacts of environmental pollutants entering the soil, including at least two aspects: human health assessment, with human health as the core goal, and ecological health assessment aimed at the stability of the soil ecosystem or its components [152]. Different countries or regions have certain differences in the starting points and steps of soil ecological risk assessment. For example, the one adopted by the United States is based on human health, and its assessment consists of three steps: problem formation, problem analysis, and risk characterization [153]. The one adopted by the United Kingdom is based on the national sustainable development strategy, and is carried out in several steps, including risk identification, consequence determination, risk perception, risk assessment, risk management, and risk monitoring [154].

China’s soil ecological risk assessment started relatively late, and experts and scholars have proposed a general method for soil ecological risk assessment in China based on the introduction and introduction of foreign theories and practical results. Risk identification, exposure analysis, and risk characterization are often used as metrics for soil ecological risk assessment [155]. Risk factor identification is the first step in soil ecological risk assessment, which lays the foundation for soil ecological risk assessment by hypothesizing the ecological effects produced by human activities, identifying risk sources, and then assessing the process. Exposure analysis is the process of using technical research on data to summarize the potential contact and symbiotic relationships between risk sources and various elements of the soil ecosystem [155]. Risk characterization is the final stage of soil ecological risk assessment, which is a comprehensive analysis of the results of the first two steps. The purpose of risk characterization is to assess the stress caused by pollutants to the soil ecosystem or some of its components through the relationship between soil pollutants and their concentrations and the resulting ecological effects, explain the risk assessment, and report the results [156].

## 5. Conclusions and Prospect

In conclusion, the future research and application of soil remediation technologies should focus on developing efficient, low-cost, and potential soil remediation technologies, such as microbial remediation and phytoremediation. The sole application of physicochemical technologies for the remediation of heavy metal-contaminated soils is not only costly but also impractical for large-scale soil improvement. Moreover, it may lead to issues such as soil structure destruction, fertility degradation, and a decline in biological activity. Phytoremediation, as an emerging and efficient bioremediation technology, has been widely applied. However, it also has drawbacks such as low biomass, strong selectivity, and the subsequent treatment of plants enriched with heavy metals after harvesting. Therefore, to address the issue of soil heavy metal pollution, it is essential to strengthen research on the ecological chemical behavior of heavy metal elements and remediation technologies, especially plant and microbial remediation technologies, which have broad application prospects. Actively exploring the combined application of various remediation technologies, leveraging their strengths and compensating for their weaknesses, will better achieve the remediation of heavy metal-contaminated soils.

It is also necessary to establish a comprehensive soil remediation assessment system to reduce the occurrence of over-remediation or under-remediation. Based on domestic and international research technologies, the development of efficient stabilization agents for the simultaneous disposal of multiple heavy metals is crucial. Additionally, interdisciplinary knowledge should be integrated to improve the efficiency of high-biomass economic plants in remediating heavy metal-contaminated soils. The establishment of heavy metal-contaminated soil remediation standards is not a simple concept or action but is based on a clear understanding of the ecological toxicological critical levels of pollutants, comprehensively considering various factors such as economic development level, social factors, political factors, clean technology level, and regulatory controls. China’s current “Soil Environmental Quality Standards” (GB15618-2018) can no longer meet the requirements of soil pollution remediation work. Formulating a new, society development-adapted, and comprehensive soil environmental quality standard is key to solving soil pollution issues at present. The remediation evaluation of heavy metal-contaminated soils is a complex process involving multiple disciplines such as ecology, ecotoxicology, environmental science, geography, soil science, and disaster science. There are significant deficiencies in both basic theoretical research and practical work, which should be paid sufficient attention in future research.

## Figures and Tables

**Table 2:** Commonly used remediation technologies for heavy metal-contaminated soils.

Remediation Technology		Mechanism	Influence Factor	Advantage	Disadvantage	References
Physical remediation	Tilling and soil replacement	Replacing or partially replacing contaminated soils with uncontaminated soils to dilute the concentration of pollutants in the soil	Quantity of soil replacement	Effectively isolating contaminated soil to reduce its impact on the environment	Labor-intensive, with high transportation costs over long distances, and only suitable for the remediation of severely contaminated soils in small areas	[53,54]
	Thermal treatment	Removing volatile heavy metals from soils by heating	The volatility of heavy metal elements in soils	It can effectively reduce the concentration of volatile heavy metals in the soil	It requires a significant amount of energy, making it unsuitable for large-scale remediation. Improper collection of heavy metals can lead to secondary pollution, and it can disrupt soil moisture and organic matter	[55]
	Solidification	Excessive power current is used to gradually heat the contaminated soils to reach the melting temperature	Temperature and soil conductivity	It is very effective in removing large amounts of heavy metal-contaminated waste and can be used for the remediation of large quantities of soils. The vitrified material can be recycled and reused as aggregates and clean fillers	It cannot be used when the soil has poor conductivity. It can only be applied to in situ solidification in moist soils with a low alkali content. The cost is prohibitive for large-scale field applications	[56]
Chemical remediation	Stabilization	Adding stabilizers to alter the physicochemical properties of the soil, or directly interacting with heavy metals through precipitation, adsorption, coordination, complexation, and redox reactions to change the form of heavy metals, thereby reducing their concentration, mobility, and bioavailability	Soil particle size and porosity, heavy metal concentration and speciation	The remediation cost is moderate, and the treatment effect is good	It does not truly achieve the removal of heavy metals; once activated, heavy metals can pollute the soil again	[57,58]
	Leaching	Using leaching solutions containing chemical reagents (acids/bases, surfactants, chelating agents, salts, or redox agents) to transfer metals from the soil into aqueous solutions	Heavy metal type, concentration, speciation, and soil property	It can completely remove heavy metals from the soil, with a high removal efficiency and easy operation	Commonly used leaching agents are either difficult to degrade or can destroy the physicochemical properties of the soil, and degradable leaching agents are relatively expensive	[59]
Bioremediation	Phytoremediation	Using chelating action, cell wall precipitation, compartmentalization, and other detoxification mechanisms of plants to absorb and concentrate heavy metals from the soil into the above-ground parts of the plants, and then harvesting the above-ground parts to achieve the removal of heavy metals	Speciation of heavy metals and soil environment	It costs the least, and the plants can be used for bioenergy production after phytoremediation, with no secondary pollution and of easy operation	It is only effective for recovering certain metals (Cd, Ni, Cu, and Zn); metal capture kinetics are very slow; metal-laden biomass must be managed after plant growth	[60,61]
	Fauna remediation	By absorbing and concentrating heavy metals themselves, thereby reducing the content of heavy metals in the soil; by improving the activation capacity of heavy metals in the soil through their own activities, promoting the enrichment of heavy metals by plants	Soil environment and speciation of heavy metals	It can improve the physicochemical properties of the soil, promote plant growth, enhance the ecological environment, and does not produce secondary pollution	Limited tolerance range, highly susceptible to environmental influences, strong specificity, and for large areas of contaminated soil, its remediation capacity may be limited	[62]
	Microbial remediation	Utilizing the adsorption, immobilization, methylation, and redox capabilities of microorganisms towards heavy metal ions, or the biopolymers produced by microorganisms to chelate or precipitate heavy metal ions, forming complexes and reducing the toxicity of heavy metals	Soil environment and temperature	Low cost, low energy consumption, capable of absorbing multiple heavy metals, reducing the remediation time, participating in the regulation of nutrient cycles in the soil, and increasing the transformation rate of heavy metals	The microbial environment is strict, and the process is difficult to control. Some microorganisms can immobilize heavy metals, reducing the absorption of heavy metals by plants	[63,64]
Combined remediation technology		Combining different remediation technologies for the remediation of heavy metal-contaminated soils	Types and speciation of soil heavy metals	It can improve the efficiency of remediation and compensate for the shortcomings of single soil remediation technologies	The application scope is limited, and current research mainly focuses on phytochemical-combined remediation and phytomicrobe-combined remediation technologies	[65,66]

**Table 3:** Soil remediation standards for heavy metal contamination in different regions.

Region	Regulations	Type of Heavy Metal	Remediation Standard (mg/kg)	Influence Factor
America	Soil Remediation Standards, 2004	Hg	270	Economic development level, social factors, clean technology factors, regulatory control factors, and political factors [128,129,130]
		Cd	100	
		Pb	600	
		As	20	
		Zn	1500	
		Cu	600	
Australia	National Environment Protection (Assessment of Site Contamination) Measure, NEPM, 1999	Hg	15	
		Cd	20	
		Pb	300	
		As	100	
		Zn	7000	
		Cu	1000	
Canada	Canadian Soil Quality Guidelines, CSQG, 1997	Hg	6.6	
		Cd	64	
		Pb	70	
		As	12	
		Zn	200	
		Cu	63	
China	Soil Environmental Quality—Risk Control Standards for Soil Pollution of Agricultural Land (Trial) (GB 15618-2018)	Hg	1.3	
		Cd	0.3	
		Pb	70	
		As	40	
		Zn	200	
		Cu	50	
Denmark	Contaminated Soil Act, 2000	Hg	1.0	
		Cd	0.5	
		Pb	40	
		As	20	
		Zn	500	
		Cu	500	
The Netherlands	Soil Remediation Circular.2006	Hg	0.3	
		Cd	0.8	
		Pb	85	
		As	29	
		Zn	140	
		Cu	36	

**Table 4:** Post-remediation monitoring and ecological assessment methods of contaminated soils.

Monitoring Methods		Principles	Ecological Assessment Steps	References
Laboratory monitoring		Ecotoxicological monitoring of remediated soil samples in the laboratory	Risk factor identification— exposure analysis—risk characterization	[135,136,137,138,139]
In situ monitoring		Performing ecological and toxicological diagnostics at the remediation site to assess the effectiveness of contaminated soil remediation		
Biochemical toxicology monitoring		Selecting indicators at the molecular and cellular levels to measure the impact of pollutants on organisms		
Ecological indication monitoring	Plant symptomatology and growth measurement method	Assess the degree of heavy metal pollution in soil through the growth status and biomass of plants		
	Sensitive animal indicator method	Assessing the degree of soil contamination by the number and survival status of sensitive animals such as earthworms in the soil		
	Biomarker method	The characterization of exposure to one or more chemical contaminants or their effects through biochemical, cellular, physiological, behavioral, or energetic changes measured in body fluids, tissues, or the whole organism		
	Soil enzyme indicator method	Evaluating remediation effectiveness by monitoring enzyme activity in soils		

## Data Availability

The data used to support the findings of this study are available from the corresponding author upon request.
